# Modeling of Photochemical Reactions in a Focused Laser Beam, II

**DOI:** 10.6028/jres.113.019

**Published:** 2008-10-01

**Authors:** Adolfas K. Gaigalas, Fern Hunt, Lili Wang

**Affiliations:** Biochemical Science Division, National Institute of Standards and Technology, Gaithersburg, MD 20899; Mathematical and Computational Sciences Division, National Institute of Standards and Technology, Gaithersburg, MD 20899; Biochemical Science Division, National Institute of Standards and Technology, Gaithersburg, MD 20899

**Keywords:** fluorescence, frequency domain, photochemical reactions, photodegradation

## Abstract

A method is described for obtaining the rate constant of the photodegradation process of fluorophores illuminated by a focused laser beam. The explicit kinetic equations, describing the population dynamics of excited singlet and triplet states, are averaged over the illuminated volume to describe the resulting fluorescence signal. The illumination is modulated at frequencies from 1 Hz to 100 Hz. Synchronous detection of the resulting fluorescence yields in-phase and quadrature components. The measurement of the ratio of quadrature to in-phase components at several power levels yields information on the photodegradation rate. Specifically it is shown that the data can be interpreted in a manner which yields the value of the photodegradation rate independently of other parameters entering the model. Experiments are performed with erythrosine B which has a large intersystem crossing rate to the triplet state. Measurements in solutions with different viscosities show that the photodegradation rate depends on the viscosity. This is interpreted as evidence for an intermolecular interaction mechanism. We explore the uncertainty of the estimated photodegradation constant taking into account the uncertainties of the measurements used in the synchronous detection technique.

## 1. Introduction

This work focuses on the photodegradation of fluorescent fluorophores in solution illuminated by a focused laser beam. The technique described in this paper may provide a quantitative method for studying laser-induced effects. There are many instances of a laser beam interacting with biological systems [1]. Examples range from laser surgery to the simple probing of labeled antibodies. In most cases the interpretation of the laser-based measurements is rather difficult. Fluorescent labels are widely used to probe cell structure and cellular events, and photodegradation is often a limiting factor. There are many difficulties in interpreting the reduction of fluorescence signal due to photodegradation. Heating, convection, and scattering are some of the confounding factors. We describe a method to obtain quantitative information about the photodegradation rate constant of fluorophores. Another example of an application that may benefit from this methodology is photodynamic therapy [2]. Our study extends the work of a previous paper [3] which presented a derivation of the kinetic equations describing photochemical reactions in a focused laser beam. This paper shows how harmonic solutions of the kinetic equation can be used to analyze experimental measurements and extract photodegradation rates.

## 2. Formulation of the Model

**Fig. 1:**
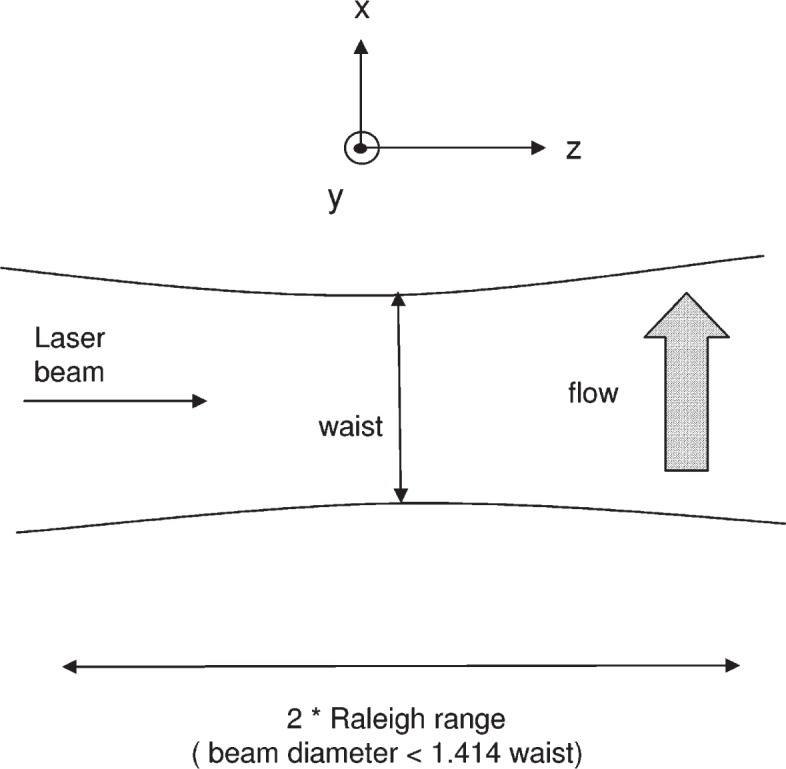
A schematic of the experimental arrangement described by the analysis. A laser beam propagates in the *z* direction (to the right) and is focused to a narrow waist. The fluorophore solution flows in the *x* direction (vertical in the figure). The detector is located along the *y* axis (perpendicular to the plane of the figure). The fluorescence is measured from a portion of the illuminated region as defined by the detector aperture.

Figure 1 shows a schematic of the physical situation under consideration in this work. A more detailed description of the apparatus is given in reference [4]. A laser beam, incident along the *z* axis, is focused to a waist of about 20 μm. A solution of fluorophores flows along the *x* axis (vertical direction in Fig. 1) and passes through the focused laser beam. A detector (not shown in Fig. 1) located along the *y* axis, which is perpendicular to the plane of the figure, detects fluorescence from the illuminated fluorophores within the acceptance aperture of the detector. The acceptance aperture should be no larger than twice the Raleigh range defined as the distance over which the beam diameter does not exceed 1.414 of the value of the minimum laser beam diameter (called the beam waist). The detector sums the photons coming from the fluorophores in the observation region. The kinetic equations that describe the temporal evolution of the fluorophore concentration are given for a three state model by

$$\begin{array}{l} \frac{\partial N_{0}(r,t)}{\partial t}+v\frac{\partial N_{0}(r,t)}{\partial x}=-k_{a}(r,t)N_{0}(r,t)+k_{r}N_{1}(r,t)+k_{T}N_{3}(r,t),\\ \frac{\partial N_{1}(r,t)}{\partial t}+v\frac{\partial N_{1}(r,t)}{\partial x}=+k_{a}(r,t)N_{0}(r,t)-k_{r}N_{1}(r,t)-k_{isc}N_{1}(r,t),\\ \frac{\partial N_{3}(r,t)}{\partial t}+v\frac{\partial N_{3}(r,t)}{\partial x}=+k_{isc}N_{1}(r,t)-k_{T}N_{3}(r,t)-k_{d}N_{3}(r,t). \end{array}$$ (1)

**Fig. 2:**
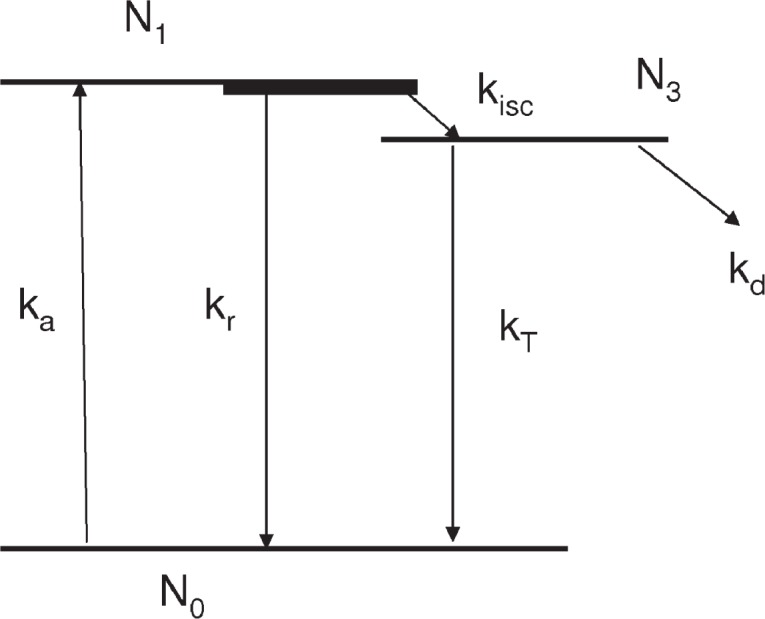
The molecular energy levels involved in the photochemical process. *N*_0_, *N*_1_, and *N*_3_ are the populations of the ground, the lowest excited singlet, and the lowest excited triplet states of the molecule. The arrows indicate the possible transitions between the states and the letters next to the arrows represent the rates of the transitions. The thick line extending the excited singlet state to the right represents the manifold of states involved in the equilibration of the initial excited singlet state. The rate *k_d_* represents all processes that result in a non fluorescent fluorophore.

The symbol *r* represents the *x*,*y* coordinates, and *ν* is the flow velocity. The various rates in Eq. (1) are defined in Fig. 2 which shows a schematic of the three state model under consideration. The radiative absorption rate is given by *k_a_*, while the total relaxation rate to the ground state is given by *k_r_*. The symbol *k_isc_* represents the transition rate between the singlet and triplet excited states. The radiative and non radiative relaxation of the triplet state are represented by *k_T_* and *k_d_* respectively. The boundary conditions are given by

$$\begin{array}{l} N_{0}(-L,y,z,t)=N^{0},\;N_{1}(-L,y,z,t)=0,\;N_{3}(-L,y,z,t)=0\;for\;t\geq 0\\ N_{0}(x,y,z,t)=N^{0},\;N_{1}(x,y,z,t)=0,\;N_{3}(x,y,z,t)=0\;for\;x\leq -L\\ N_{1}(x,y,z,0)=0,\;N_{3}(,x,y,z,0)=0\;for\;x\geq -L \end{array}$$ (1a)

The first two lines of Eq. (1a) state that prior to entering the laser beam located at *x* = 0, the fluorophores are in the ground state with a concentration given by *N*^0^. The third line of Eq. (1a) states that prior to turning on the laser at *t* = 0, the excited state populations are zero. *L* is a distance beyond which the laser intensity vanishes. The inclusion of the triplet state provides a more realistic model of the photodegradation process than the two state model. The absorption rate, *k_a_*, shown in Fig. 2 is the only rate constant which varies with time and position. The solution of the extended model will follow that given previously for the two state model [3]. Briefly, Eq. (1) is multiplied by a constant *ε* = 10^−8^ and it is noted that the factors *k_r_ε* and *k_isc_ε* are of the order 1, while *k_a_*(*r,t*)*ε* is of the order 1 over most of the illuminated volume; *k_T_ε* is of the order 10^−3^ and *k_d_ε* is of the order 10^−6^. Therefore it is convenient to look for solutions to Eq. (1) which are correct to different orders in *ε*. Specifically, we write the solutions to Eq. (1) as

$$\begin{array}{l} N_{0}(x,y,t,\varepsilon )={\hat{N}}_{0}(x,y,t)+\varepsilon {\hat{N}}_{01}(x,y,t)+O(\varepsilon^{2})\\ N_{1}(x,y,t,\varepsilon )={\hat{N}}_{1}(x,y,t)+\varepsilon {\hat{N}}_{11}(x,y,t)+O(\varepsilon^{2})\\ N_{3}(x,y,t,\varepsilon )={\hat{N}}_{3}(x,y,t)+\varepsilon {\hat{N}}_{31}(x,y,t)+O(\varepsilon^{2}). \end{array}$$ (2)

Inserting Eq. (2) into Eq. (1) and collecting different orders of *ε*, we obtain for the zero order the relations shown as

$$\begin{array}{l} {\hat{N}}_{1}(r,t)=\frac{k_{T}}{k_{isc}}{\hat{N}}_{3}(r,t)\\ {\hat{N}}_{0}(r,t)=\frac{k_{T}(k_{r}+k_{isc})}{k_{a}(r,t)k_{isc}}{\hat{N}}_{3}(r,t). \end{array}$$ (3)

Next, the total number of intact fluorophores is defined by

$$N(r,t)\equiv {\hat{N}}_{0}(r,t)+{\hat{N}}_{1}(r,t)+{\hat{N}}_{3}(r,t).$$ (4)

Thus to the first order in *ε*, the kinetic equation for *N*(*r,t*) is given by

$$\frac{\partial N(r,t)}{\partial t}+v\frac{\partial N(r,t)}{\partial x}=-k_{d}{\hat{N}}_{3}(r,t).$$ (5)

Eq. (3) and Eq. (4) are used to rewrite ${\hat{N}}_{3}(r,t)$ in terms of $\hat{N}(r,t)$ and the resulting expression for ${\hat{N}}_{3}(r,t)$ is inserted into Eq. (5). The result is given by Eq. (6) where in addition we assumed that *k_T_* ≪*k_isc_*, and terms of the order $k_{T}^{2}$ have been dropped.

$$\frac{\partial N(r,t)}{\partial t}+v\frac{\partial N(r,t)}{\partial x}=-k_{d}\frac{k_{a}(r,t)k_{isc}}{k_{T}(k_{r}+k_{isc})(1+\frac{k_{a}(r,t)k_{isc}}{k_{T}(k_{r}+k_{isc})})}N(r,t).$$ (6)

Using the same notation as in reference [3] define

$$\begin{array}{c} k_{a}(r,t)=\sigma P_{c}P(r,t)\\ b=\frac{\sigma P_{c}}{k_{T}(1+\frac{k_{r}}{k_{isc}})}\\ \eta =k_{d}b \end{array}$$ (7)

Where *P*(*r,t*) is the laser power, *σ* (cm^2^) is the molecular absorption cross section, *P*_c_ = *λn*/*hc* where *λ* is the wavelength, *n* is the index of refraction, *h* is Planck constant, and *c* is the speed of light in vacuum. Inserting the above definitions into Eq. (6), we obtain the final form of the kinetic equation.

$$\frac{\partial N(r,t)}{\partial t}+v\frac{\partial N(r,t)}{\partial x}=-\frac{\eta P(r,t)}{1+bP(r,t)}N(r,t)$$ (8)

Equation (8) is identical to Eq. (14) in Ref. [3]. The averaging of Eq. (8) over the observation volume follows the same procedure as shown previously [3]. The resulting kinetic equation for the number of intact fluorophores integrated over the entire observation region, 〈***N***(*t*)〉, is reproduced below [3].

$$\frac{d}{dt}\langle N(t)\rangle =\frac{-\eta \alpha (P_{0}+\Delta (t))}{1+b\alpha (P_{0}+\Delta (t))}\langle N(t)\rangle -\frac{v}{1.274\cdot w}\cdot \frac{\left(1+b\alpha^{\prime }(P_{0}+\Delta (t))\right)}{1+b\alpha (P_{0}+\Delta (t))}(\langle N(t)\rangle -N^{0})$$ (9)

where

$$\Delta (t)=P_{1}\cos(\omega t)\text{and}\;\langle N(t)\rangle =\iint_{R}f(x,y)N(x,y,t)dxdy.$$

Here *P*_1_ is the amplitude of modulated laser power, *P*_0_ is the constant laser power, *f* (*x,y*) is the spatial profile of the laser beam, and *ω* = 2 *πf*, the frequency of modulation. The other terms in Eq. (9) are *ν*, the velocity of flow, *w*, the width of the laser beam, and *b*, and *η* are defined in Eq. (7). The quantities *a* and *a′* depend on the beam profile function *f* (*x,y*) which is assumed to be a Gaussian function with area normalized to 1. Explicitly

$$\alpha \equiv \frac{\int_{R}f(x,y)f(x,y)N(x,y)dxdy}{\int_{R}f(x,y)N(x,y)dxdy}.$$ (10)

The value *α*′ a of is calculated using Eq. (10) with *N*(*x,y*) replaced by the derivative ∂*N*(*x,y*)/∂*x*. It was shown in a previous paper [3] that to a very good approximation the values of *α* and *α*′ are time independent when *η* is small, and that they can be calculated using the time independent, steady state solutions of the complete set of kinetic equations. Consequently we use the time independent solution, *N*(*x,y*), in Eq. (10). The spatial profile of the laser beam is described by the function [5]

$$\begin{array}{l} f(x,y)=\frac{1}{\pi w^{2}}\exp(-\frac{x^{2}+y^{2}}{w^{2}})\\ P_{0}(x,y)=P_{0}f(x,y)\\ \Delta P(x,y,t)=P_{1}f(x,y)\cos(\omega t). \end{array}$$ (11)

The fluorescence signal originates from the radiative relaxation of molecules excited by the laser beam. The fluorescence signal from any location in the laser beam depends on the laser power and the concentration of fluorophores at that location. An explicit form of the fluorescence signal integrated over the entire observation region can be derived by an averaging argument. The result is given by Eq. (12) and is accurate for small laser power levels.

$$F(t)=B^{\prime }k_{rad}b\left[P(t)\langle N(t)\rangle -b\alpha {\left(P(t)\right)}^{2}\langle N(t)\rangle \right].$$ (12)

Here *B′* is an instrument constant, *k_rad_* is the radiative decay rate of the molecular excited state and *P*(*t*) = *P*_0_+ *P*_1_cos (*ωt*) ≡ *P*_0_ + Δ(*t*). The fluorescent signal can therefore be obtained (with a small error) by solving a single ordinary differential equation, Eq. (9), for 〈***N*** (*t*)〉, and then using the solution in Eq. (12). Together these equations represent an experimentally accessible mathematical model of the fluorescent signal due to excitation by a focused laser beam. The model can be used to analyze measurements. The problem is that the two equations contain many parameters, *η*, *b*, *α*, *α*′, *v*, and *w*, and it is not clear how to extract the value of the relevant physical quantity, *k*_d_.

## 3. Proposed Measurement Procedure

### 3a. Derivation of the Harmonic Measurement Model

Consider the case of very small laser power. We make the approximation, *bαP*(*t*) << 1, *bα*′*P*(*t*) << 1, and write Eq. (9) as

$$\begin{array}{l} \frac{d}{dt}\langle N(t)\rangle +\left[\eta \alpha (P_{0}+P_{1}e^{j\omega t})+\frac{v}{1.274\cdot w}\right]\\ \;\langle N(t)\rangle =\frac{v}{1.274\cdot w}\cdot N^{0}. \end{array}$$ (13)

Where *j* is the imaginary number, and we have written the modulated laser power as a complex quantity, *P*(*t*) = *P*_0_ + *P*_1_*e^jωt^*. By writing the time dependent power modulation as *P*_1_*e^jωt^* (*P*1 real) and assuming a complex solution, we are solving the complex kinetic equations simultaneously for two power modulations: *P*_1_ cos (*ω*t) and *P*_1_ sin (*ω*t). The solution to Eq. (13) is then represented by the coefficient of *e^jωt^*. In the case where the response follows the sinusoidal modulation without any lag, the response will be given by *N*e*^jωt^* where *N* is a real number. If the response of the system lags the modulation then there will be a measurable phase lag between the sinusoidal excitation and the sinusoidal response. In this case the solution will be given by *N*e*^jωt^* where *N* is a complex number. The complex number *N* can be written as |*N*|*e^jθ^* where |*N*| is the magnitude and *θ* is the phase difference between the driving modulation and the complex solutions. The phase is calculated using θ=tan−1(Im(N)Re(N)) where Im(*N*) is the imaginary part of the complex number and Re(*N*) is the real part. For small values of the phase, *θ* = Im(*N*)/Re(*N*)(< 4 % error for *θ* < 17°). Since the phase lag is approximately given by the ratio of the imaginary and real parts of the solutions, the calculation of the phase is very simple. The mathematical economy of this method is the motivation for using a complex modulation. Since the modulation contains a constant component, *P*_0_, the solution is also assumed to have a constant component, *N*_0_, and a time dependent response, *N*_1_. The explicit assumed solution has a complex Fourier expansion given by

$$\langle N(t)\rangle =N_{0}+N_{1}e^{jwt}$$ (14)

where we will ignore terms of higher frequencies because they do not contribute to the measured fluorescence response. The procedure for finding the solution, 〈*N*(*t*)〉, is to insert Eq. (14) into Eq. (13) and set equal the terms on each side that have the same time variation. Carrying out the prescribed procedure, the time independent part and the part that varies as *e^jωt^* are given by

N0=AN0A+ηαP0=AN0BN1=−ηαN0P1B+jω=−N0ηαP1BB2+ω2+jN0ηαPω1B2+ω2≡NRe+jNIm. (15)

The constant *A* is defined by *A* = *v*/1.274*w*, and the constant B is defined by *B* = *A* + *ηaP*_0_. According to Eq. (12), for very small power levels the integrated fluorescence signal is proportional to *P*(*t*)〈*N*(*t*)〉. The fluorescence response is obtained by inserting the expression for power of the laser, *P*(*t*), and the solution 〈*N*(*t*)〉 given by Eq. (15), yielding the expression for the complex magnitude of the fluorescence signal: *N*_0_*P*_1_ + *N*_Re_*P*_0_ + *jN*_lm_*P*_0_. After some algebra, we obtain the ratio of the imaginary and real part of the fluorescence signal given by

imaginaryreal=NImP0N0P1+NReP0=ηαωP0A(A+ηαP0)+ω2. (16)

The form of Eq. (16) suggests a possible interpretation of the measurements. An approach is to measure the ratio of the imaginary and real parts of the response as a function of frequency for several power levels *P*_0_. At each power level it is possible to fit the frequency dependence to a function of the form *a*_1_*ω*/(*a*_2_ + *ω*^2^) and obtain the value of the parameter *a*_1_. According to Eq. (16), the parameter value should depend linearly on power with the slope equal to *ηα* = *k_d_ bα*. From the slope it is possible to determine the product of the decay rate, molecular rate constants, and a property of the laser beam. This is not very satisfactory because the result does not provide unique information about the photodegradation rate.

Consider next the case of an larger power level. Repeating the steps leading to Eq. (15) and using the entire Eq. (9) leads to the following solution

$$\begin{array}{l} N_{0}=\frac{A(1+b\alpha^{\prime }P_{0})}{\eta \alpha P_{0}+A(1+b\alpha^{\prime }P_{0})}N^{0}\\ N_{1}=\frac{-\eta \alpha \frac{1}{\left(1+b\alpha P_{0})(1+b\alpha^{\prime }P_{0}\right)}N_{0}P_{0}}{\frac{\eta \alpha P_{0}+A(1+b\alpha^{\prime }P_{0})}{\left(1+b\alpha P_{0}\right)}+j\omega }. \end{array}$$ (17)

Applying this solution to calculate the fluorescence response as given by Eq. (12), we obtain the ratio of the imaginary part and the real part given by

$$\frac{imaginary}{real}=\frac{\frac{\eta \alpha P_{0}}{\left(1+b\alpha P_{0})(1+b\alpha^{\prime }P_{0}\right)}\omega }{\frac{\eta \alpha P_{0}+A(1+b\alpha^{\prime }P_{0})}{\left(1+b\alpha P_{0}\right)}A(1+b\alpha^{\prime }P_{0})+\omega^{2}}.$$ (18)

**Fig. 3:**
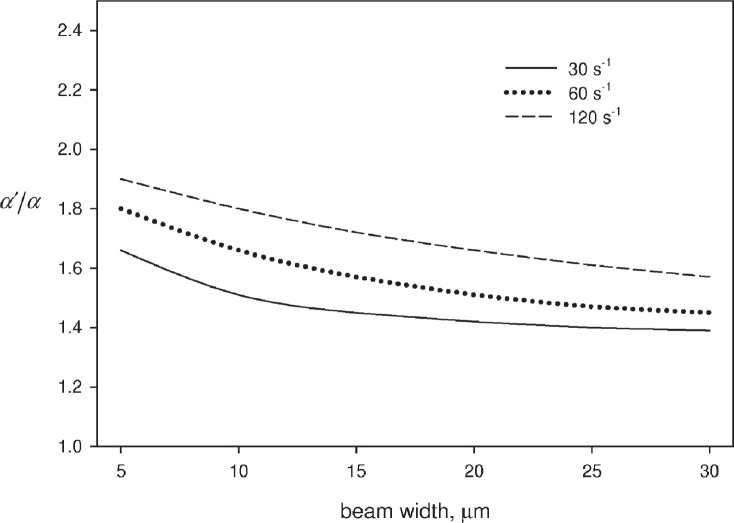
The dependence of the ratio *α*′/*α* on the beam width and the photodegradation rate constant. The three lines (dashed, dotted, and solid) correspond to the indicated values of the photodegradation rate. The calculation is performed using the steady state solutions of the kinetic model. The calculation shows that the ratio, *α*′/*α*, varies relatively little when photodegradation rate varies by a factor of four.

At first glance this result does not look promising. However, the denominator is to a good approximation dominated by the term *A*^2^ + *ω*^2^ and the ratio of *α* and *α*′ is approximately a constant for a given laser beam geometry. This ratio can be calculated by Eq. (10). Figure 3 shows the value of *α* and *α*′ as a function of beam width for three values of the photodegradation rate, *k*_d_. The beam width is one half of the beam waist. In the first approximation, the quantities *α* and *α*′ are related to each other by a constant factor of about 1.45. For specific beam width of 20 μm the value of the ratio ranges from 1.42 to 1.66 depending on the value of the photodegradation rate constant. In absolute terms, a range of 300 % variation in the photodegradation rate constant results in a 14 % variation in the ratio *α*′/*α*. Thus it is reasonable to set the value of the ratio *α*′/*α* to a constant in Eq. (18) and proceed to evaluate the photodegradation rate constant. This is demonstrated in the following.

### 3b. Proposed Measurement Procedure

Equation (18) suggests that the ratio of quadrature to the in-phase components of the measured response can be fitted to a function of modulation frequency of the form

$$\frac{imaginery}{real}=\frac{a_{1}\omega }{a_{2}+\omega^{2}}.$$ (19)

Measurements carried out at different values of power provide a dependence of the parameter *a*_1_ on laser power. Equation (18) suggests that the measured parameter *a*_1_ obtained from the frequency dependence of the response should depend quadratically on the laser power. Hence it should be possible to extract two parameters from the fit of the dependence of *a*_1_ on power. Setting the ratio *α*′/*α* equal to 1.44, the explicit functional form of the dependence of *a*_1_ on power is given by

$$a_{1}=\frac{cP_{0}}{\left(1+dP_{0})(1+d1.44P_{0}\right)}.$$ (20)

The two parameters are *c* = *ηα* as in the case of low power, and the additional parameter *d* = *bα*. The ratio of these two parameters gives the photodegradation rate. What is the origin of this happy fact? The variation of *a*_1_ on laser power has two sources, first due to the photodegradation process described by parameter *c*, and second due to the saturation of the excited triplet state population described by parameter *d*. The reduction due to photodegradation involves the same molecular optical parameters as the reduction due to saturation. Therefore in the ratio of the two fit parameters, *c*/*d*, the optical parameters cancel out leaving only the photodegradation rate. The final result is summarized by

$$k_{d}=\frac{c}{d}.$$ (21)

A practical consequence of Eqs. (20) and (21) is that the absolute power level of the laser beam does not have to be measured. Using the absolute power level or the relative power in Eq. (20) simply changes the magnitude of the two parameters *c* and *d*, however the ratio of the two parameters remains the same in both cases.

In the following section, measurements will be presented of the photodegradation of erythrosine B in order to demonstrate the validity of the proposed measurement technique.

## 4. Measurement of the Photodegradation Rate of Erythrosine B

**Fig. 4:**
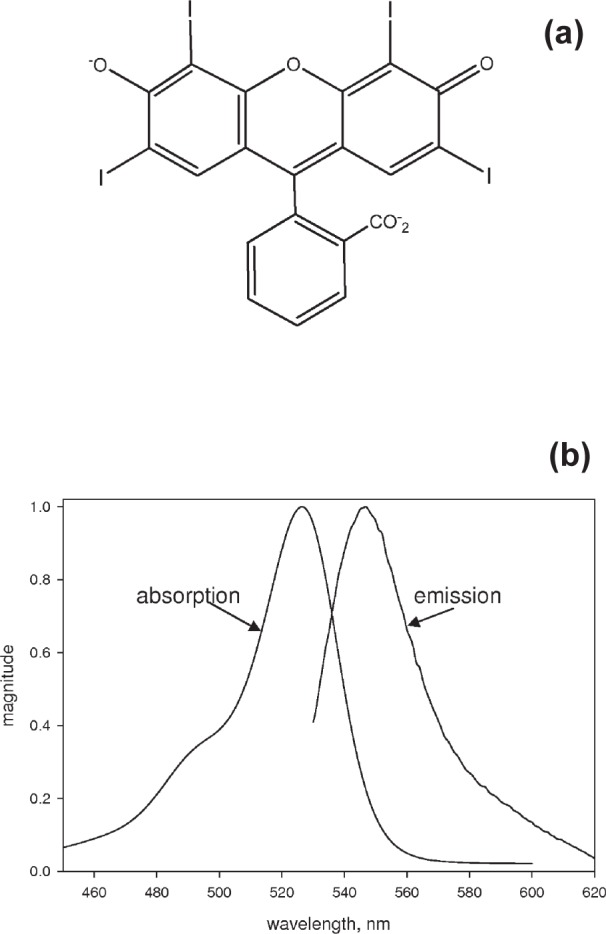
(a) The chemical structure of erythrosine B. The fluorophore is obtained by replacing four hydrogen atoms in fluorescein by iodine atoms. The heavy iodine atoms increase the transition rate between the singlet and triplet excited states. (b) The normalized absorption and emission spectra of erythrosine B in phosphate buffer saline (PBS). The fluorescence emission is insignificant beyond the wavelength of 620 nm.

Figure 4a shows the chemical structure of erythrosine B, a derivative of fluorescein with four hydrogen atoms replaced by iodine atoms. The heavier iodine atoms increase the rate of transitions from the excited singlet state to the slightly lower first excited triplet state. Recent measurements have shown that erythrosine B exhibits phosphorescence at 700 nm (relaxation of the triplet state), and delayed fluorescence which occurs due to backward transitions from the triplet to the singlet excited state and subsequent radiative relaxation of the singlet state [6]. The emission spectrum, shown in Fig 4b, did not show significant intensity in the 700 nm region. Measurements were performed below 25 °C to minimize delayed fluorescence which becomes significant only at elevated temperature (> 30 °C) [7]. The fluorescence intensity from erythrosine B depends non linearly on the intensity of the incident illumination. This dependence was interpreted as a depletion of the ground state population due to the saturation of the triplet excited state[7–9]. The large triplet state population and the quenching of the triplet state by dissolved oxygen has been utilized in the design of dissolved oxygen sensors[10]. Because of the large triplet state population, erythrosine B is a good candidate for demonstrating the fluorescence modulation technique.

**Fig. 5:**
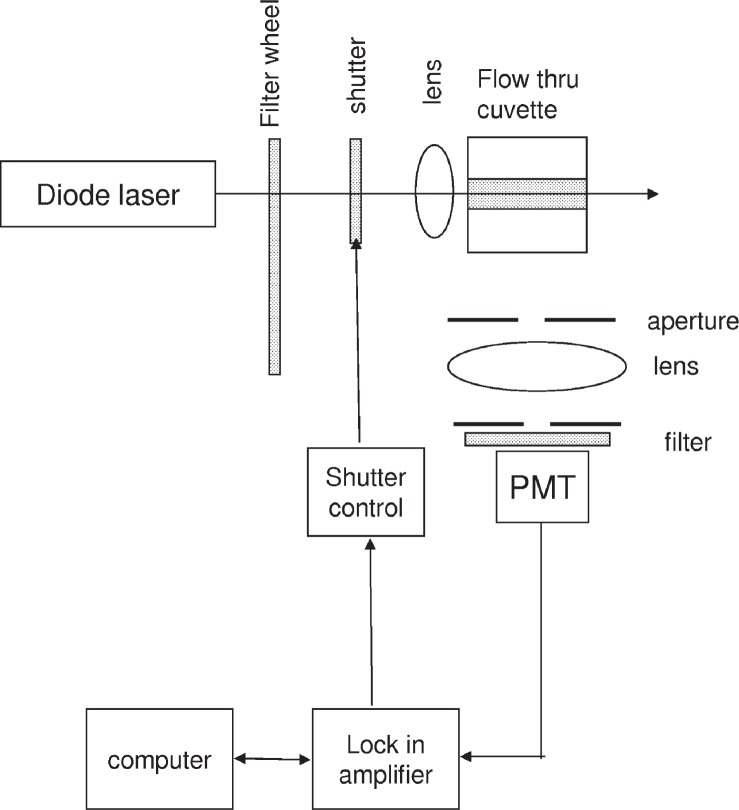
A schematic of the apparatus used for the measurement of the photodegradation rate. The flow thru cuvette is viewed from the top with the flow coming out of the paper (*x* axis in Fig. 1). The laser beam passes thru the cuvette along the long dimension of the flow channel. The filter wheel contains ND filters of increasing OD, and the shutter modulated the laser intensity with a frequency controlled by the lock-in amplifier internal reference oscillator. The signal detected by the PMT is routed to the lock-in amplifier which gives the in-phase and quadrature components of the signal.

**Fig. 6:**
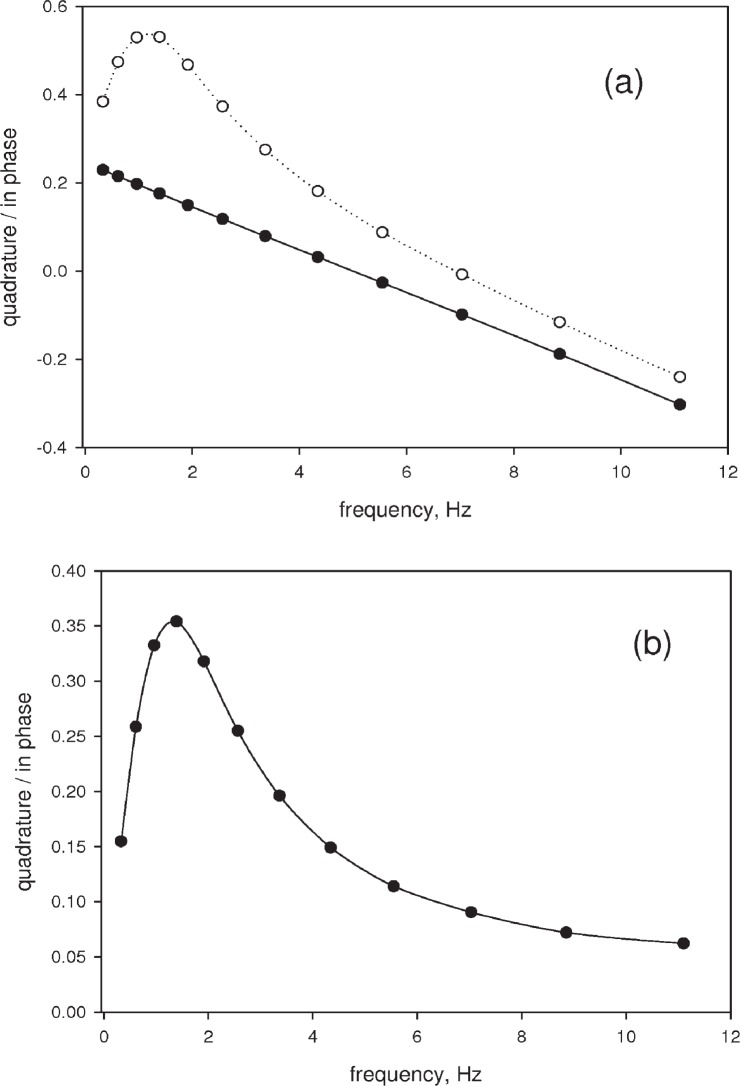
(a) The solid circles show the ratio of the quadrature component and the in-phase component of the measured scattered signal as a function of the modulation frequency. Since scattering is an instantaneous event the measured phase is due to the response of the instrument. The open circles show the ratio of the quadrature component and the in-phase component of the measured fluorescence signal as a function of the modulation frequency. The fluorescence response is delayed relative to the illumination modulation resulting in additional phase shift. (b) Subtracting the instrumental phase shift (solid circles in Fig. 6a) from the fluorescence phase shift (open circles Fig. 6a) gives the phase shift due to the dynamics of the fluorescence process alone. The solid curve in Fig. 6b is a spline fit to the data showing the trends in the data.

**Fig. 7:**
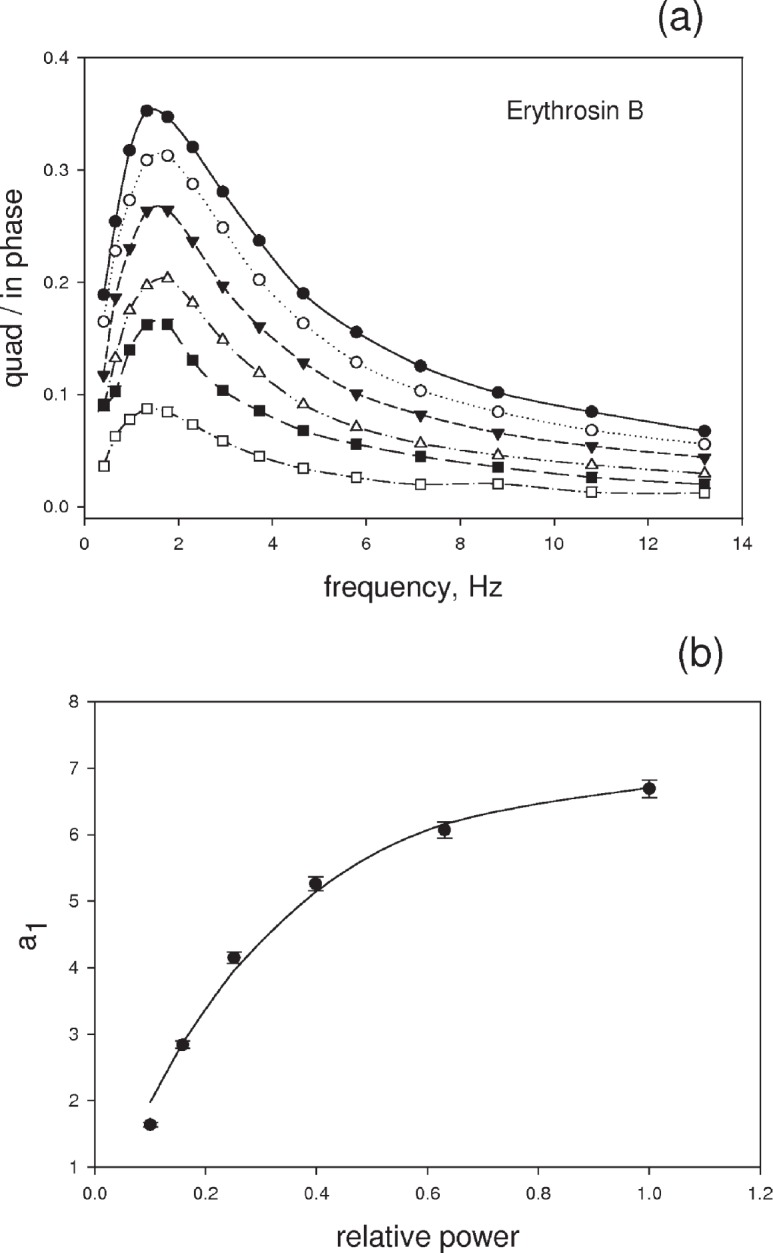
(a) The six sets of symbols give the response at six different laser power levels. The response at each power level is fitted to the expected response given by *a*_1_*ω*/(*a*_2_ + *ω* ^2^) to obtain the parameter *a*_1_. (b) The solid circles show the dependence of *a*_1_ on the relative power level of the incident laser light. The error bars associated with the values of *a*_1_ were obtained from the fitting procedure used in Fig. 7a. The solid curve is a best fit to Eq. (20) discussed in the text. The ratio of the fit parameters gives the photodegradation rate.

A schematic of the measurement apparatus is shown in Fig. 5 which shows the apparatus from the top. A diode laser (Ventus 532 System, Laser Quantum Ltd, Stockport, UK)^1^ serves as the source of illumination. The laser beam passes through a filter wheel containing a set of neutral density (ND) filters with the optical density (OD) varying from 0 to 1 in increments of 0.2. Turning the filter wheel provides a means to reproducibly vary the laser power. The laser itself was run at a indicated current of 60 mA corresponding to a nominal power output of about 30 mW. After passing the filter wheel, the laser beam strikes a mechanical shutter (CH-60, Boston Electronics, Brookline, MA) that provides the modulation of the laser beam intensity. Finally, the laser beam is focused by a 4X objective in a flowing solution contained in a flow thru cuvette. The cuvette flow channel has a cross section of 10 mm by 4 mm, and the laser beam propagates in the *z* direction defined as that parallel to the 10 mm side of the cuvette. The flow is out of the plane of Fig. 5 (defined as the *x* direction). The photomultiplier detector (PMT), placed along the *y* direction, views the illuminated portion of the solution through a lens, a band pass filter, and two apertures. The first aperture controls the amount of light collected and the second aperture (placed at the focal plane) determines the part of the illuminated solution which is observed. The band pass filter, centered at 560 nm, ensures that only erythrosine B fluorescence is detected. The signal from the PMT is routed to a current amplifier (SR570, Stanford Research Systems, Stanford, CA) and finally to a lock in amplifier (SR 830 DSP Lock-In, Stanford Research Systems, Stanford, CA). The lock-in amplifier is controlled by a computer which sets the lock-in internal reference frequency and reads the resulting PMT signal. A TTL output, locked to the internal reference signal of the lock-in amplifier and located on the rear panel of the lock-in amplifier, is used to energize the shutter controller. The time constant of the lock-in amplifier was set to 1 s. During the acquisition of the signal, a time delay was placed between the setting of the lock-in reference frequency and the reading of the PMT signal. The delay was set to the time it took for the lock-in amplifier to settle after a change of reference frequency. The acquisition of signal consisted of 20 sequential “reads” of the in-phase and quadrature components. The ratio of the quadrature and in-phase components was calculated and presented as the phase. Thus the phase presented in the following figures is the average of the 20 “reads”. The standard deviation of the 20 samples was much smaller than the size of the circles in the plots presented in Figs. 6a and 7a. Twelve frequencies were chosen such that the logarithm of the frequencies was spaced equally. This resulted in more points at low frequencies where the response variation was the greatest. A 35 mL capacity syringe pump (Harvard Apparatus 22, South Natick, MA) was used to pump the solution through the cuvette. At the low flow rates used for the measurements, six frequency scans were performed each for a different ND filter wheel setting. A constant solution flow was maintained throughout the six frequency scans.

The open circles in Fig. 6a show a typical response for erythrosine B solution for a filter wheel set to OD = 0. The solid circles in Fig. 6a show the response from a sintered plate of glass replacing the fluorophore solution. The scattering from glass is an instantaneous process and it should not lead to any phase shift in the observed signal relative to the excitation. Therefore the observed phase shift is due to the behavior of the instrumentation. The shutter has a fixed opening and closing time interval, therefore the shape of the light pulse will change with frequency resulting in an instrumental “apparent” phase shift. The instrumental phase shift was highly reproducible, and it was always subtracted from the phase shift observed for the fluorophore solution, resulting in a phase shift which depended only on the fluorophore population dynamics. As an example, the phase shift resulting from the subtraction of the two measured phase shifts in Fig. 6a is shown in Fig. 6b by the solid circles. The solid curve in Fig. 6b is a spline curve used to show the trend in the data.

Figure 7a shows a typical set of six frequency scans taken at decreasing laser power for erythrosine B in a PBS solution. The instrumental phase shift was subtracted out in all cases. The responses in Fig. 7a were fitted to Eq. (9) and the value of parameter *a*_1_ was obtained as a function of laser power (see Eq. (20). The solid circles in Fig. 7b show the dependence of the parameter *a*_1_ obtained from the data in Fig. 7a on laser power. The uncertainties in the values of the parameters are of the size of the solid circles and are indicated by the error bars. The uncertainty estimates were obtained from the fitting algorithm used in the SigmaPlot 9 application (Systat Software, Inc., San Jose CA). The solid curve in Fig. 7b is a fit to Eq. (20). The deviations between the data and the fit are small (< 5 %) yielding good estimates of the parameters *c* and d in Eq. (20). The ratio of the two parameters gives a photodegradation constant of (33 ± 2) s^−1^ where the uncertainty is discussed in Sec. 5. An important point is that the power axis in Fig. 7b is given in terms of relative power obtained from the value of the OD filter. Using the absolute power levels would change the values of the fit parameters but the ratio of the parameters would remain the same. This suggests that the value of the absolute power of the laser beam is not critical in obtaining the photodegradation rate. There are systematic errors in the analysis inherent in the assumption that the ratio *α*′/*α* is a constant. The present analysis suggests that the systematic errors are relatively small.

The measurements were repeated in solutions of increasing viscosity. The viscosity was progressively increased by mixing in 1 % by mass dextran (a polysaccharide) or 1 % by mass PEO (polyethylene oxide) long chain polymers in the buffer containing erythrosine B. The response and analysis of the fluorescence measurements were similar to those shown in Fig. 7a and Fig. 7b. Table 1 shows the measured photodegradation rates in the increasingly viscous solutions. The fact that the photodegradation rate decreases with increasing viscosity suggests that the underlying reaction is diffusion limited. This would be the case if erythrosine B reacted with the photoproduct resulting from the relaxation of the triplet state. For example singlet oxygen can be produced by a reaction between the dissolved triplet oxygen and the excited triplet state of erythrosine B. Once produced, the singlet oxygen can react with erythrosine B and alter its structure. The role of diffusion in the chemical reaction is on the microscopic level and does not affect the assumptions made previously that the role of diffusion is negligible in macroscopic mass transfer into the entire illuminated region. The dependence of photodegradation rate on viscosity (and hence on diffusion) suggests the approach can be used to study the mechanism of the photodegradation process. The photodegradation rate can be measured for a set of environmental conditions that are deemed important. The variation of the photodegradation rate with the environmental parameters can suggest a model for the photodegradation process. This strategy was followed previously by relating the decrease in the photodegradation rate of fluorescein to the increase of the concentration of *n*-propyl gallate, a known singlet oxygen quencher [11].

## 5. Uncertainty Analysis

The photodegradation rate is given by the ratio of two parameters, *c* and *d*, in Eq. (20). The parameters are obtained by fitting the function containing the two parameters to the values of the parameter *a*_1_ obtained for different values of the relative laser power. The values of *a*_1_ are in turn obtained by fitting the function containing *a*_1_ Eq. (19) to the measured ratios of quadrature and in phase fluorescence signals at different modulation frequencies. The uncertainties of the two measured fluorescence signals are set equal to the standard deviation of several repetitive measurements taken under identical conditions. Given the uncertainties of the quadrature and in phase signals, the uncertainty in the ratio of the two signals can be obtained by using a simple error propagation formula. In theory, the best fit to the ratio of the signals, using the uncertainties as weights, gives values of *a*_1_ with uncertainties which take into account both the uncertainties in the ratio of the signals and the fitting process. The uncertainties in *a*_1_ would in turn be used as weights in the fitting to obtain the parameters *c* and *d*. In theory, the final uncertainties of *c* and *d* would depend on the uncertainties of the quadrature and in phase signals plus the additional uncertainties introduced by the two fitting procedures. In practice, the SigmaPlot algorithm used in the fitting does not use uncertainties of the data in the fitting algorithm. Rather the algorithm estimates the parameter uncertainties by examining the deviations between the unweighted data and the best functional fit. The uncertainties presented in Table 1 are the uncertainties given by the fitting algorithm used to obtain the parameters *c* and *d*.

## 6. Conclusion

The fluorescence response of fluorophores in solution illuminated by a modulated laser beam can be measured using synchronous detection with a lock-in amplifier. The ratio of the quadrature to the in-phase components of the measured response can be fitted to a function of the modulation frequency given by *a*_1_*ω*/(*a*_2_ + *ω*^2^). The fitting yields values of the parameters *a*_1_ and *a*_2_. In this paper we show how the analytical solution of a mathematical model of the experiment (derived in an earlier paper [3]) allows the expression of the parameters *a*_1_ and *a*_2_ in terms of the physical constants of the experiment. The measurement can be repeated for several power levels resulting in a measured dependence of the parameter *a*_1_ on the power of the laser. The dependence of the parameter *a*_1_ on the power can be fitted to a function, *a*_1_ = *cP*_0_/(1 + *dP*_0_) (1 + *d*1.44*P*_0_), where 1.44 is a property of the laser beam geometry corresponding to the beam width. The ratio of the two fitting parameters, *c*/*d*, gives the photodegradation rate *k_d_*. The ratio is not sensitive to uncertainties in the absolute power level of the laser. The utility of this measurement method is based on the separation of the photodegradation rate from all of the optical transitions rates associated with the molecular excited states.

## Figures and Tables

**Table 1:** 

Solvent	*k_d_* = *c*/*d*, s^−1^
PBS buffer	33 ± 2
PBS buffer + 1 % dextran	28 ± 2
PBS buffer + 1 % PEO	20 ± 2

## Footnotes

1 Certain commercial equipment, instruments, or materials are identified in this paper to foster understanding. Such identification does not imply recommendation or endorsement by the National Institute of Standards and Technology, nor does it imply that the materials or equipment identified are necessarily the best available for the purpose.
